# Effects of biological monitoring and results outreach on private landowner conservation management

**DOI:** 10.1371/journal.pone.0194740

**Published:** 2018-04-04

**Authors:** Seth H. Lutter, Ashley A. Dayer, Emily Heggenstaller, Jeffery L. Larkin

**Affiliations:** 1 Department of Fish & Wildlife Conservation, Virginia Tech, Blacksburg, Virginia, United States of America; 2 Indiana University of Pennsylvania Research Institute, Indiana, Pennsylvania, United States of America; 3 Department of Biology, Indiana University of Pennsylvania, Indiana, Pennsylvania, United States of America; 4 American Bird Conservancy, The Plains, Virginia, United States of America; Oregon State University, UNITED STATES

## Abstract

Sustained management efforts by private landowners are crucial for the long-term success of private land natural resource conservation and related environmental benefits. Landowner outreach is a primary means of recruiting private landowners into voluntary conservation incentive programs, and could also help sustain conservation behaviors through time. However, evaluation of outreach targeting landowners during or after participation in natural resource conservation incentive programs is lacking. We assessed two methods of landowner outreach associated with a Natural Resources Conservation Service incentive program targeting effective management of early successional forest habitat on private land in the Appalachians and Upper Great Lakes regions of the United States. While early successional forest habitat benefits many wildlife species, the program target species were the Golden-winged Warbler (*Vermivora chrysoptera*) and American Woodcock (*Scolopax minor*). After habitat management through the program occurred, biological technicians monitored wildlife and vegetation on enrolled properties and results were communicated to landowners in mailed packets. Our research focused on whether landowner interactions with technicians or receipt of result mailings could influence landowner post-program management intentions and management-related cognitions (e.g., agency trust, perceptions of outcomes). We conducted a telephone survey with landowners from January to May 2017, and analyzed survey data using quantitative group comparisons and qualitative coding methods. Landowners that accompanied biological technicians on monitoring site visits had higher agency trust and more positive perceptions of program outcomes. Result mailings did not improve landowner perceptions of program outcomes or agency trust, but did provide benefits such as increased landowner knowledge about birds. Neither outreach method was associated with more positive landowner post-program management intentions. Our findings underline the importance and potential of direct interactions between conservation biologists and landowners. These two forms of non-traditional outreach administered by biologists could be a worthwhile component of future conservation program evaluations on private lands.

## Introduction

Natural resource conservation on privately owned lands is critically important for the protection of biodiversity and ecosystem services in the United States and around the world [1]. With greater than 70 percent of the contiguous United States held under private ownership, private landowner cooperation is fundamental for achieving goals such as wildlife habitat conservation on a landscape scale [2, 3]. Private land conservation takes many forms, from the establishment of conservation easements to active management approaches such as buffer strip installation or sustainable timber harvests. In the United States, federal conservation programs funded by the Farm Bill (Agricultural Act of 2014) and administered by agencies such as the U.S. Department of Agriculture’s Natural Resources Conservation Service (NRCS) are the largest funding source for private land conservation [3]. These programs provide financial and technical assistance to enable landowners to conduct conservation practices that benefit individual landowners, society, and the environment [4].

Outreach is a central tool used to encourage private landowners to undertake conservation, through participation in federal programs or otherwise. Conservation related outreach includes many forms of communication and stakeholder engagement techniques, such as educational programs, personal contacts, and informational mailings [5]. The purpose of most conservation related outreach is to influence the cognitions or behaviors of a target audience [6]. Research has demonstrated the importance of outreach for influencing private land conservation behaviors. For example, landowners with access to quality information and familiarity with agency personnel are more likely to use best management practices [7]. Relationships with agency staff and one-on-one agency visits can also encourage landowners to participate in conservation programs [8]. For landowners who are already participants in voluntary conservation programs, communication and contact with agency staff contributes to landowner satisfaction [9] and continued use of conservation practices [10].

Interactive, personal methods of communication are recognized as the most effective means of conservation outreach [11] and conservation agencies such as the NRCS acknowledge the importance of personalized interactions with landowners [12]. However, limited funding for staff and technical assistance are barriers for federal agencies in the United States to communicate consistently and proactively with private landowners [13]. Separate from these challenges, the NRCS-led Conservation Effects Assessment Project (CEAP) was initiated in 2004 to help quantify environmental benefits of federal conservation programs. The CEAP effort relies on diverse partnerships with non-governmental science and technology partners to implement outcome-based monitoring and assessment projects. In addition to quantifying environmental outcomes, monitoring initiatives such as those supported by CEAP may provide an avenue for outreach to landowners involved in conservation management. The process of biological monitoring on private land has some basic elements suitable for landowner outreach. Biological monitoring tends to require landowner interactions through site visits and related scheduling, and produces site-level information that could be of interest to landowners. However, it is unclear if monitoring-associated outreach to these already committed landowners could improve program experiences or influence future management behaviors.

In the Eastern United States, NRCS incentive programs targeting effective management of early successional forest habitat provide an opportunity to examine how outreach to landowners can shape social outcomes of conservation program participation. The long-term decline of early successional forest habitat and associated wildlife species such as Golden-winged Warbler (*Vermivora chrysoptera*) and American Woodcock (*Scolopax minor*) is a major conservation issue [14, 15]. ‘Early successional forest habitat’, hereafter referred to as ‘young forest’, is habitat with persistent shrubs or seedling to sapling-sized trees. This successional habitat is typically caused by disturbance events such as timber harvest, wind-throw, or fire [16]. A key feature of young forest habitat is the inherent need for recurring management such as timber harvests to create new young forest or maintain shrublands, and retain habitat quality for associated wildlife species [17]. In addition to this necessity for continued management, past human dimensions research has shown that landowners most likely to manage for young forest are those who have already done so in the past [18]. Thus, these landowners are an important group for creating new young forest and maintaining this habitat on the landscape. There is also high potential for outreach to influence this group of landowners. Research has found that policy tools such as financial incentives and educational outreach would be most influential among landowners who had already conducted young forest management in the past [19].

Elements of the NRCS Working Lands for Wildlife effort and the Regional Conservation Partnership Program provide incentives to create young forest on private lands in the Appalachians and Upper Great Lakes regions of the United States. Young forest habitat benefits many species of wildlife, but these two program applications are specifically aimed at providing habitat for Golden-winged Warblers and American Woodcock. An ongoing CEAP assessment is studying the biological effectiveness of these NRCS efforts in terms of vegetation and bird response to management actions [20, 21]. The CEAP monitoring process involves two methods of outreach: biological technician site visits and communication of site-specific monitoring results to landowners. This outreach could build landowner commitment for continued management. Although not a complete substitute for traditional visits from agency or extension staff, site visits from biological technicians are an opportunity to engage landowners in-person, build relationships, and provide scientific information about monitored properties [22]. In this CEAP assessment, biological technicians were affiliated with the Indiana University of Pennsylvania or the American Bird Conservancy. Communication of monitoring results constituted an additional, complementary approach to site visits for the CEAP assessment. Giving individual landowners feedback on the environmental benefits of their management actions has been suggested as one way to encourage continued use of conservation practices [23]. This feedback strategy has some basis in existing interventions designed to alter conservation-related behaviors. For example, in the field of home energy conservation, mailed feedback on household performance has been shown to cause significant, lasting reductions in homeowner energy consumption [24].

Evaluating whether biological monitoring related outreach could influence landowner conservation management intentions was our primary interest. Landowner post-incentive program management, either through program re-enrollment or behavioral persistence without further incentives, is important for long-term conservation outcomes on the land [25]. To assess outreach efficacy, we also considered other cognitive elements that may serve as behavioral antecedents. Drawing from literature on landowner conservation behaviors we identified several social variables that are likely to facilitate the effects of outreach on landowner behavior. These cognitive variables included landowner perceptions of management outcomes, agency trust, program satisfaction, outcome beliefs, and normative beliefs.

Perceptions have been broadly defined as “the way an individual observes, understands, interprets and evaluates a referent object… or outcome” [26]. In the case of natural resource conservation programs, positive or negative perceptions of management effects could be influenced by outreach. For example, landowner perceptions of successful management are related to follow-up communication from habitat program biologists [27], and landowner perceptions of conservation practice effectiveness are related to the use of those practices [28, 29].

Trust is an important component of many natural resource management contexts [30]. Several dimensions have been used to conceptualize trust including rational trust, affinitive trust, and procedural trust [30]. Each of these dimensions of landowner trust in the NRCS and agency partners could be influenced by effective outreach efforts. Rational trust (rooted in evaluations of expertise and utility; 30) and affinitive trust (based on emotional connections and feelings of shared values; 30) in particular could be bolstered by agency interactions and feedback on management successes.

Landowner satisfaction can stem from fulfilled participation motivations [9, 31]. Outreach that helped meet landowner motivations could generate satisfaction with the program and the sponsor agency. Satisfaction has been shown to relate to continued conservation efforts, especially through continued program participation [9].

Outcome beliefs are assessments of the likely outcomes of future behaviors, and are thought to drive attitudes toward specific behaviors and behavioral intentions [32]. A landowner who believes a management action will result in positive and desired outcomes would be expected to be more likely to implement that action (e.g., 18). Outreach that highlights the positive effects of management could encourage a landowner to think future management is likely to result in positive outcomes as well.

Landowner normative beliefs about conservation management relate to social pressures to use a management practice. Important normative beliefs include whether other people perform a behavior (descriptive norms) and whether others approve or disapprove of the behavior (injunctive norms) [32]. Landowner beliefs about management norms have been shown to influence management intentions [33] and could be positively affected by outreach. Interaction with technicians who promote conservation or messages that emphasize collective achievements could change landowner normative beliefs related to management practices.

Drawing on these variables, our research investigated how two methods of outreach—biological technician site visits and monitoring result mailings—influenced landowners in two young forest habitat conservation programs. Specifically, we hypothesized that result mailings and technician site visits would increase landowners’ post-program management intentions and improve management-related cognitions including perceptions of management outcomes, agency trust, program satisfaction, outcome beliefs, and normative beliefs.

## Methods

### Biological monitoring and results communication

Our study population consisted of 189 landowners that signed conservation program contracts with NRCS between 2012 and 2016 to manage for young forest on properties in Maryland, Minnesota, New Jersey, Pennsylvania, and Wisconsin. After management began, these landowners voluntarily allowed biological technicians onto their properties to monitor for birds and vegetation regrowth as part of the CEAP assessment. At the time of biological monitoring site visits the managed properties were either under a current NRCS contract or had recently finished a contract with NRCS to create young forest. The monitoring process involved 4–5 site visits to a managed property between mid-April and mid-July each year in 2015 and/or 2016. In total each property was visited 1–4 times to survey American Woodcock, 2–4 times to survey songbirds including the Golden-winged Warbler, and 1–2 times to survey vegetation. Technicians notified landowners prior to each site visit. The extent of landowner-technician interactions varied among landowners. Some landowners never met with technicians, some greeted technicians at the property, and others accompanied technicians during the site visit(s).

Using biological data collected from monitored properties, we summarized bird response to habitat management efforts in site-specific result mailing packets (S1 Appendix) for each landowner. Property visitation dates and detection numbers for the two target species (Golden-winged Warbler and American Woodcock) were detailed explicitly. A list of all bird species that were detected on the landowner’s property was also included. Species of Greatest Conservation Need (as defined by associated State Wildlife Action Plans, e.g., [34]) were marked with an asterisk in these lists. Results were carefully worded to emphasize the positive effects of management while accurately conveying the monitoring data from the landowner’s property. The mailing concluded with encouragement to continue to create and maintain young forest. Past research on effective landowner communications in this context was incorporated into the mailings, such as the term ‘young forest’ rather than the term ‘early successional habitat’ [35] and a focus on young forest management benefits for wildlife [18]. The mailings also referenced the collective accomplishment of landowners in the program and concluded with encouragement to continue to create and maintain young forest habitat.

### Survey design

We developed a telephone survey questionnaire to explore post-program management intentions, perceptions of program outcomes, program satisfaction, agency trust, outcome beliefs, and normative beliefs. Social scientists at Virginia Tech, cooperating NRCS staff, and private lands biologists reviewed the survey. The survey was pre-tested with 8 private landowners who participated in similar NRCS habitat conservation programs. The survey consisted of primarily closed-ended questions. Only survey items used in analyses reported in this manuscript are discussed here (see S2 Appendix for full survey). We restricted analysis to those measures that were hypothesized to be affected by outreach efforts.

The independent variables in this study related to the two outreach methods being investigated. Both were operationalized as binary variables. Biological technician site visits were operationalized as whether or not a landowner had accompanied a technician during at least one site visit—a level of interaction expected to have the greatest impact for a landowner. The other independent variable—result mailing reception—was whether or not a landowner had received a result mailing with monitoring results.

The dependent variables related to post-program young forest management intentions and management-related cognitions. Landowner intentions to manage for young forest within 10 years after their NRCS contract by re-enrolling in an NRCS program (S2 Appendix, #13) or if further cost share was not available (#17) were measured on 5-point Likert-type scales from ‘not at all likely’ to ‘extremely likely’. A list of landowner motivations for owning woodland from the National Woodland Owner Survey [36] was adapted into a set of potential motivations of participating in the habitat program. For each motivation a follow-up item (#9A, 9C-9I) asked what effect program participation had for that related program outcome, on a 5-point Likert-type scale from ‘very negative effect’ to ‘very positive effect’. Satisfaction with the habitat program overall, cost share payments, wildlife outcomes, and interactions with NRCS (#11A-D) were measured on a 1 to 10 scale, from ‘not at all satisfied’ to ‘completely satisfied’. Agency trust was operationalized using three items (#12A-C) corresponding with three dimensions of trust (affective, rational, and procedural trust).

Outcome beliefs about the effects of future management were measured with 7 items (#19A-G), which corresponded to the 8 perception items (access to expert advice was not considered a relevant outcome of future management and was therefore excluded). Landowner normative beliefs about nearby landowners were measured with two items (#20A, C) relating to descriptive norms (whether nearby landowners manage for young forest), and injunctive norms (whether nearby landowners think the respondent should manage for young forest). Another item (#20B) looked at normative influence–the importance of nearby landowners’ opinions to the respondent. Injunctive norms of people important to the respondent were measured with two items (#21A-B).

We also developed a shortened follow-up survey questionnaire as a post-test for a sub-set of landowners after they received result mailings. The follow-up survey included repeated measures of all of the items outlined above and concluded with one additional open-ended question that assessed landowner thoughts on the effect of the result mailings: “What effect, if any, did the result mailing have on you?” Responses were recorded in the data file verbatim, and then read back to landowners to ensure accuracy.

### Survey methods

Between January 2017 and May 2017, we conducted the primary survey with all landowners in the study population. To maximize the response rate, we varied the day of week and time of day that we attempted to reach landowners via phone. If two phone numbers were available in the database for a landowner we tried both. We left no more than two messages on a landowners’ voicemail or with another person who answered the line. Survey responses were entered into Qualtrics software.

At the time of the primary survey, 63.4% (n = 120) of the population had been sent result mailings in October 2015 and again in December 2016. The other group of landowners had received no result mailings at the time of the primary survey, and served as a pseudo-control group. The pseudo-control landowner group was sent result mailings in April 2017 after completing the primary survey. The follow-up survey was then conducted in May 2017 with landowners in the pseudo-control group who had completed the primary survey and indicated interest in the follow-up.

This research was conducted with approval from, and in accordance with, the Virginia Tech Institutional Review Board (Protocol #16–597). Before completing the telephone survey, respondents were read a consent statement informing them of the study’s purpose and confidentiality of their responses. Respondents were then asked to provide oral consent stating their agreement to participate in the survey. Verification that the respondent had expressed verbal consent was recorded in the data file. Written consent was not obtained due to the telephone survey methodology, and the Virginia Tech Institutional Review Board approved the oral consent procedure. Members of the research team also signed compliance agreements that ensure NRCS cooperators will not disclose protected agricultural or personally identifiable information, as required by Section 1619 of the Food, Conservation, and Energy Act of 2008.

Of the 189 landowners contacted for the primary survey, 102 completed the survey for a response rate of 57.9%. The primary survey took 30 minutes on average to complete. For the follow-up survey, 32 of the 42 eligible landowners completed the survey for a response rate of 76.2%.

To check for non-response bias in terms of contract characteristics, group comparisons (Mann-Whitney U and chi-square tests) between primary survey respondents and non-respondents were made using contract data in the CEAP assessment database. The variables used for non-response tests included ‘years since contract start’, ‘acres planned’, ‘property region’, and ‘practices contracted’. Practices used by 10 or fewer landowners total were dropped from comparisons to ensure adequate sample sizes for statistical tests. The only significant difference detected (*χ*^2^ = 5.095, *p* = 0.024) was for the practice ‘Tree/Shrub planting’ (one of nine contracted practices), which a greater proportion of respondents conducted (10.8%) than non-respondents (2.4%). The sample was not weighted to adjust for this minor difference.

### Analysis

We analyzed our data using SPSS (version 24.0). Incomplete questions from completed surveys were dropped on an analysis-by-analysis basis. One scale was constructed using the mean of two items that measured injunctive norms of important others (#21A-B). The Cronbach’s alpha for this scale was 0.78, indicating a high degree of reliability.

Shapiro-Wilk tests were used to assess response normality, and two sets of Mann-Whitney U tests were used to analyze the primary survey data. The first set of Mann-Whitney U tests compared the variables of interest for landowners who received result mailings and landowners who had not received result mailings. The second set of tests compared landowners who accompanied biological technicians on at least one site visit and landowners who had not. In order to correct for running multiple independent comparisons, Benjamini-Hochberg values [37] were utilized to assess p-value significance with a false discovery rate of 5%. The two sets of Mann-Whitney U tests were treated separately for these corrections.

We paired primary survey responses to each respondent’s follow-up survey responses to further examine the effect of the results mailing on these individuals. Wilcoxon signed-rank tests were used to compare primary survey and follow-up survey responses, using the same set of survey items as the dependent variables in the Mann-Whitney U tests. Benjamini-Hochberg values were also used to assess significance for these paired comparisons.

Our qualitative data analysis included responses from the 32 respondents who completed the follow-up survey in May 2017 and answered the open-ended question “What effect, if any, did the result mailing have on you?” We created a comprehensive and mutually exclusive code list based upon major recurring response themes and coded responses accordingly. Some respondents discussed more than one theme, so individual responses were often coded for multiple themes.

## Results

### Primary survey

Survey respondents were primarily male (88%) and averaged 61 years old (median = 63 years, SD = 11 years). The majority (66%) had a four-year college degree or higher. Respondents owned their land for an average of 37 years (median = 20 years, SD = 35 years), and owned a mean of 780 acres (median = 235 acres, SD = 2133 acres). Respondents’ enrolled properties were located in Pennsylvania (59%), Minnesota (30%), New Jersey (7%), Maryland (2%), and Wisconsin (2%).

About a third (36%) of respondents lived within one mile of the property enrolled in the habitat program. The remaining 64% of respondents lived greater than one mile from the enrolled property, and were considered absentee landowners [36]. Chi-square tests detected no significant associations between absentee status and result mailing reception (*χ*^2^ = 1.516, *p* = 0.218) or absentee status and whether a landowner had accompanied a technician (*χ*^2^ = 3.146, *p* = 0.076).

Of 102 surveyed landowners, 33 reported accompanying biological technicians on at least one site visit of their property and 69 did not accompany technicians. Several Mann-Whitney U tests (Table 1) detected significant differences between landowners who accompanied biological technicians on at least one site visit and landowners who did not accompany technicians.

**Table 1:** Comparison of phone survey responses of landowners in NRCS young forest habitat programs based on whether they accompanied technicians monitoring enrolled properties for birds and vegetation post-management, Eastern United States, February- June 2017.

Variable	Mean		Z (U)*	p -value
	Did Not Accompany Technician (n = 69)	Accompanied Technician (n = 33)		
*Young forest management intentions*				
Program re-enrollment	3.74	3.91	0.78 (1243.5)	0.434
Management if further cost share not available	3.12	3.48	1.51 (1324)	0.130
*Perceptions*: *Effect of participation on…*				
Access to expert advice	4.25	4.70	3.20 (1538)	**0.001**
Hunting opportunities	3.83	3.97	0.89 (1255)	0.372
Bird-watching opportunities	3.88	4.48	3.51 (1571)	**<0.001**
American Woodcock	3.76	4.04	1.70 (717)	0.088
Golden-winged Warbler	3.87	3.86	0.00 (493.5)	1.000
Other birds that use young forest	4.33	4.63	1.90 (990.5)	0.058
Scenery	3.48	3.73	0.83 (1251.5)	0.404
Forest health	4.30	4.59	2.03 (1182)	0.042
*Satisfaction*				
Overall program satisfaction	8.59	9.00	1.41 (1324)	0.159
Cost share satisfaction	8.51	9.00	1.18 (1292.5)	0.240
Wildlife outcome satisfaction	8.00	8.36	0.61 (1168.5)	0.543
NRCS satisfaction	8.90	9.52	2.26 (1415.5)	0.024
*Trust*				
Rational trust	4.45	4.52	0.62 (1213.5)	0.536
Affinitive trust	4.42	4.72	2.59 (1413.5)	**0.010**
Procedural trust	4.31	4.61	1.84 (1349)	0.066
*Outcome Beliefs*: *Managing for young forest within ten years after the contract would…*				
Benefit hunting opportunities	4.48	4.91	2.63 (780)	**0.009**
Benefit bird-watching opportunities	4.39	4.88	3.52 (907.5)	**<0.001**
Benefit American Woodcock	4.18	4.35	1.32 (758)	0.189
Benefit Golden-winged Warbler	4.20	4.60	2.77 (825)	**0.006**
Benefit other birds that use young forest	4.45	4.81	2.82 (952.5)	**0.005**
Improve the scenery	3.96	4.32	1.73 (751)	0.084
Benefit forest health	4.43	4.70	2.10 (911.5)	0.035
*Normative Beliefs*				
Descriptive norm: nearby landowners	1.98	1.78	1.48 (869)	0.140
Injunctive norm: nearby landowners	1.93	2.30	-0.74 (536)	0.457
Normative influence: nearby landowners	2.85	2.31	-2.39 (423.5)	0.017
Injunctive norm: important people	3.59	3.39	-0.60 (646)	0.547

Bolded p-values are significant with Benjamini-Hochberg correction procedure for multiple independent comparisons.

*Mann-Whitney U Test Statistic.

Landowners who accompanied technicians had more positive perceptions of program participation effects on their access to expert advice and bird-watching opportunities. Those landowners who accompanied technicians also believed in the benefits of future young forest management for hunting, bird-watching, Golden-winged Warblers, and other birds more strongly than those who had not accompanied technicians. Landowners who had accompanied technicians also had higher affinitive agency trust. We found no significant differences between the two groups in terms of future management intentions or satisfaction with program components.

Of 102 surveyed landowners, 58 landowners had received the result mailings and 44 had received no result mailings (pseudo-control group). A chi-square test detected no significant association between landowners who had accompanied biological technicians and landowners who had received result mailings (*χ*^2^ = 1.91, *p* = 0.167). Mann-Whitney U tests detected no significant differences between landowners who had received the result mailings and landowners who had received no result mailings (pseudo-control group) in terms of management intentions, perceptions of program outcomes, or outcome beliefs (Table 2). We detected no significant differences in other key measures including satisfaction, trust, and normative beliefs.

**Table 2:** Comparison of phone survey responses of landowners in NRCS young forest habitat programs based on reception of mailing with bird monitoring results, Eastern United States, February- June 2017.

Variable	Mean		Z (U)*	p -value
	Received No Mailing (n = 44)	Received Mailing (n = 58)		
*Young forest management intentions*				
Program re-enrollment	3.91	3.71	-1.00 (1134.5)	0.319
Management if further cost share not available	3.23	3.25	0.71 (1264)	0.944
*Perceptions*: *Effect of participation on…*				
Access to expert advice	4.41	4.38	-0.82 (1167.5)	0.412
Hunting opportunities	3.84	3.90	-0.76 (1265.5)	0.939
Bird-watching opportunities	3.93	4.19	1.15 (1402)	0.250
American Woodcock	3.88	3.84	-0.20 (597)	0.843
Golden-winged Warbler	3.93	3.83	-0.61 (508.5)	0.543
Other birds that use young forest	4.35	4.50	0.59 (907)	0.558
Scenery	3.64	3.50	-0.91 (1145)	0.361
Forest health	4.51	4.33	-1.28 (855)	0.201
*Satisfaction*				
Overall program satisfaction	8.77	8.69	-0.84 (1159.5)	0.403
Cost share satisfaction	8.80	8.57	-0.85 (1158.5)	0.397
Wildlife outcome satisfaction	8.17	8.09	-1.25 (1026)	0.212
NRCS satisfaction	9.02	9.16	-0.24 (1244.5)	0.808
*Trust*				
Rational trust	4.55	4.41	-1.33 (1105)	0.183
Affinitive trust	4.57	4.47	-0.95 (1133.5)	0.345
Procedural trust	4.44	4.38	-0.73 (1152)	0.465
*Outcome Beliefs*: *Managing for young forest within ten years after the contract would…*				
Benefit hunting opportunities	4.56	4.66	0.07 (702)	0.947
Benefit bird-watching opportunities	4.59	4.53	-0.56 (643.5)	0.577
Benefit American Woodcock	4.47	4.07	-2.44 (495.5)	0.015
Benefit Golden-winged Warbler	4.42	4.28	-1.16 (574)	0.248
Benefit other birds that use young forest	4.65	4.52	-0.87 (706)	0.387
Improve the scenery	4.17	4.02	-0.70 (595.5)	0.491
Benefit forest health	4.68	4.40	-1.83 (633)	0.068
*Normative Beliefs*				
Descriptive norm: nearby landowners	1.89	1.92	0.52 (564)	0.603
Injunctive norm: nearby landowners	2.29	1.87	-1.66 (634.5)	0.097
Normative influence: nearby landowners	2.72	2.60	-0.65 (575)	0.516
Injunctive norm: important people	3.67	3.41	-0.87 (674.5)	0.382

Bolded p-values are significant with Benjamini-Hochberg correction procedure for multiple independent comparisons.

*Mann-Whitney U Test Statistic.

### Follow-up survey

No significant differences were detected by Wilcoxon signed-rank tests comparing landowners’ responses before and after receiving a result mailing (the same set of survey items were used as for the previous Mann-Whitney U tests). Seven themes emerged through qualitative analysis of landowner responses to the open-ended question in the follow-up survey (Table 3).

**Table 3:** Qualitative analysis of follow-up phone survey responses to “What effect, if any, did the result mailing have on you?” by landowners in NRCS young forest habitat programs, Eastern United States, May-June 2017.

Thematic Code	Frequency (%)	Definition	Example Response
Bird knowledge	23 (71.9%)	Landowner learned about birds on their property from the mailing	“We learned stuff we didn't know about the land and what’s on it. They listed a bunch of birds they recorded on the property that a lot of us didn't know were there.”
Satisfied	18 (56.3%)	Landowner was generally happy with the mailing or felt good about the results	“The membership liked receiving the info and were happy to know what was in it.”
Social interactions	10 (31.3%)	Landowner mentioned sharing the mailing with others or interactions with biologists and technicians	“Since I got grandkids to share it with them and my son and daughter to let them know since it will be their land someday.”
Management effects	9 (28.1%)	Landowner indicated an improvement on their property related to young forest management	“It was really a positive letter, made me feel a lot better about the mess out there, that the birds are arriving and will continue to arrive, especially the warbler.”
Motivated	6 (18.8%)	The mailing motivated landowner to take actions such as looking for birds or continued management	“Encouraged me to continue to manage for young forest.”
Reinforced observations	6 (18.8%)	The mailing matched or reinforced landowner’s personal observations on their property	“We've seen an increase in birds, turkeys, different animals we've never seen before. There were a lot of trees before so it is easier to see now.”
Negative	2 (6.3%)	Landowner felt negatively about the mailing or the results from their property	“I was disappointed, I should have known once the trees were gone other species would go too.”

The most commonly occurring theme in the open-ended responses was ‘bird knowledge’, which related to landowners learning about bird diversity and bird presence on their property. The second most common theme, ‘satisfied’, included responses indicating that the landowner was generally satisfied with the mailing or felt positively about the results. Responses coded as the ‘social interactions’ theme mostly expressed that the result mailing had been shared with family, friends, or neighbors. Some responses with this theme also indicated that the landowner had positive interactions with technicians during the monitoring process. The ‘management effects’ theme included responses that connected young forest management with changes on the respondent’s property, such as an increase in wildlife numbers or diversity. Responses with the ‘motivated’ theme mentioned how the mailing motivated the respondent to take action, either to look for birds on their property or to continue management for young forest. The ‘reinforced observations’ theme was associated with responses indicating that the mailing matched with the landowner’s personal observations of wildlife or forest health on their property. The least common theme was ‘negative’, corresponding to responses that were negative about the mailing and/or the property results.

## Discussion

Our results suggest an important difference in efficacy between the two forms of landowner outreach evaluated in this study. Biological monitoring technician interactions with landowners (in the form of landowners accompanying technicians on site visits) were related to a range of positive social outcomes for landowners. In contrast, result mailing communications had limited effects on landowners. Our results are comparable to other research that has demonstrated personal, interpretive outreach is more influential than passive forms of outreach [38]. Additionally, neither outreach method in our study was associated with higher landowner intentions to manage for young forest after the conservation program. The potential complexity and costs of young forest management are factors that could easily dampen the influence of a positive program experience, which may explain why outreach was not as effective in this respect. These findings align with research in the environmental education field, which has shown that information alone is not sufficient to change behaviors [39]. Pairing informative mailings with other interventions such as personal interactions and signs can be effective at achieving behavioral changes [40]. However, we were unable to examine interactive effects between the result mailing and technician interactions because of sample size. A separate variable that might have an influence on outreach effects is ownership status as either a resident or absentee landowner. Sample size limited us from investigating interaction effects between outreach and landowner absentee status.

Landowners that accompanied technicians had higher affinitive trust for NRCS and partners, a dimension of trust based on feelings of shared values and connectedness that can result from positive shared experiences [30]. Better perceptions of program outcomes and more positive outcome beliefs about future management were also associated with accompanying technicians. While these significant differences match our hypotheses based upon prior research findings, we are unable to assume the causal effect of technician interactions. For example, landowners more interested in bird-watching may have been more likely than non-birders to take an opportunity to look for birds on their property. It is possible that landowners accompanied technicians to supervise a visit to their property, rather than due to a high interest in birds. Exposure to the tangible benefits of management and interactions with a scientific expert during a site visit could be a powerful interactive experience for landowners. Our findings suggest that site visits and direct interactions had an important, positive influence on landowners. These contracted technicians helped landowners see their managed properties in a more positive light and provided a relatable face for the NRCS even though they are not NRCS staff. For these NRCS programs and others, partner positions and contractors make up a significant portion of landowner contacts, and may play a key role in shaping landowner experiences with conservation programs and perceptions of the sponsor agency.

The result mailings were beneficial in several respects; they increased landowner knowledge about birds on their property, increased landowner satisfaction to some extent, provided an interesting item to share with family and friends, and inspired a few landowners to observe birds or manage for young forest. However, our results suggest that few landowners made causative connections between management actions and effects for birds on their land. Surprisingly, two landowners also responded negatively to the mailings. While all mailings contained similar positive messages and lists of detected bird species, many landowners learned that there were no detections for one or both target species on their land, which could explain these few negative results. The uncertainty of detecting positive results in the form of species presence from conservation projects in the short-term is a possible risk of giving monitoring results feedback. Further, neither the primary or follow-up survey found significant differences in future management intentions based on result mailing receipt. Overall the mailings were not effective at encouraging future management or changing landowner cognitions. Yet, it may still be worthwhile to incorporate this element into biological monitoring strategies when easily communicated data are collected. Providing feedback on management efforts can be an incentive for landowners to allow technicians onto their properties for monitoring projects and help build relationships for ongoing monitoring purposes [22].

## Conclusions

Relationship-building between landowners and agency staff is an important and often overlooked component of conservation programs. In cases of limited or absent agency staff capacity, biologists on monitoring contracts with a resource agency may fill an agency surrogate role for productive landowner interactions. If landowners are interested and contexts are favorable, instructing biological technicians to take landowners on monitoring site visits could help build favorable perceptions of both management practices and the sponsor agency, and possibly commitment to continued management. In lieu of direct interactions with other conservation professionals, biological technicians can provide positive personal interactions that may keep landowners engaged. Our results emphasize the importance of having biological technicians with the ability and passion to effectively communicate with and educate landowners. As the final contacts many landowners have with conservation programs, monitoring technicians have an opportunity to leave a favorable last impression that promotes future management behaviors. Providing technicians with training on how to interact with landowners could increase the likelihood of these positive impressions. Although technician interactions may be a beneficial supplement, this is not a panacea outreach solution. Technicians may not have the same expertise or capacity for long-term relationship-building as professionals at natural resource agencies, university extension offices, and conservation NGOs that make contact with landowners as part of existing positions.

Alterations to our result mailing design could potentially increase the effectiveness of results communication. Changes could involve a greater emphasis on the efficacy of the collective landowner effort and accomplishment, comparisons to other landowners in the program, the inclusion of pre- and post-management data, and additional reminders (e.g. signs, bumper stickers) of the management behavior. Including measures related to other management outcomes, such as game species abundance or habitat diversity, could also broaden the appeal of results to landowners. We recommend that future research examine interaction effects between different outreach methods such as in-person interactions and informational mailings. It would also be informative to track landowners throughout the life of conservation program contracts to understand how landowners are affected by program participation and interactions with agency and partner staff, contractors, and biological technicians. A longitudinal approach could better evaluate the causal role of outreach on landowner outcomes and explore the mechanisms for how different forms of outreach bring about positive effects. More generally, we also recommend that agencies contracting out efforts such as post-management monitoring consider the importance of contractor interactions with landowners, and more explicitly encourage partners to purposefully implement landowner outreach well. With limited resources available for landowner outreach, it is essential that those interactions with landowners that do take place are effective at encouraging conservation efforts.

## Supporting information

S1 Appendix Result mailing example. (PDF)

S2 Appendix Primary telephone survey questionnaire. (DOCX)

S1 Dataset Survey dataset. (SAV)
