# Supplementary material for: Effects of biological monitoring and results outreach on private landowner conservation management
Source: PLoS One. 2018 Apr 4;13(4):e0194740. doi: 10.1371/journal.pone.0194740 (PMC5884542; doi:10.1371/journal.pone.0194740)
Supplement: S1 Appendix — (PDF) [file pone.0194740.s001.pdf]

YOUR RESULTS ARE IN!

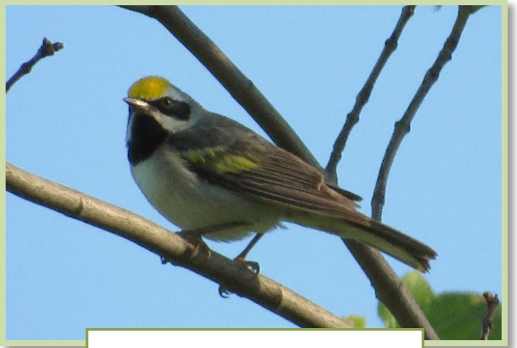

Golden-winged Warbler

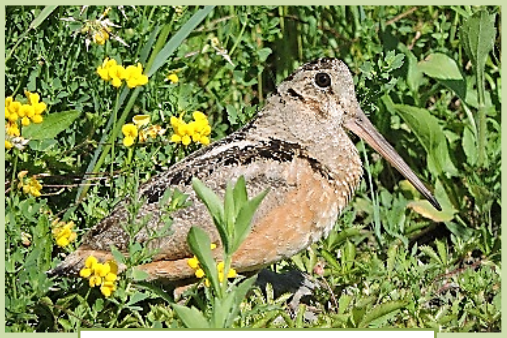

American Woodcock

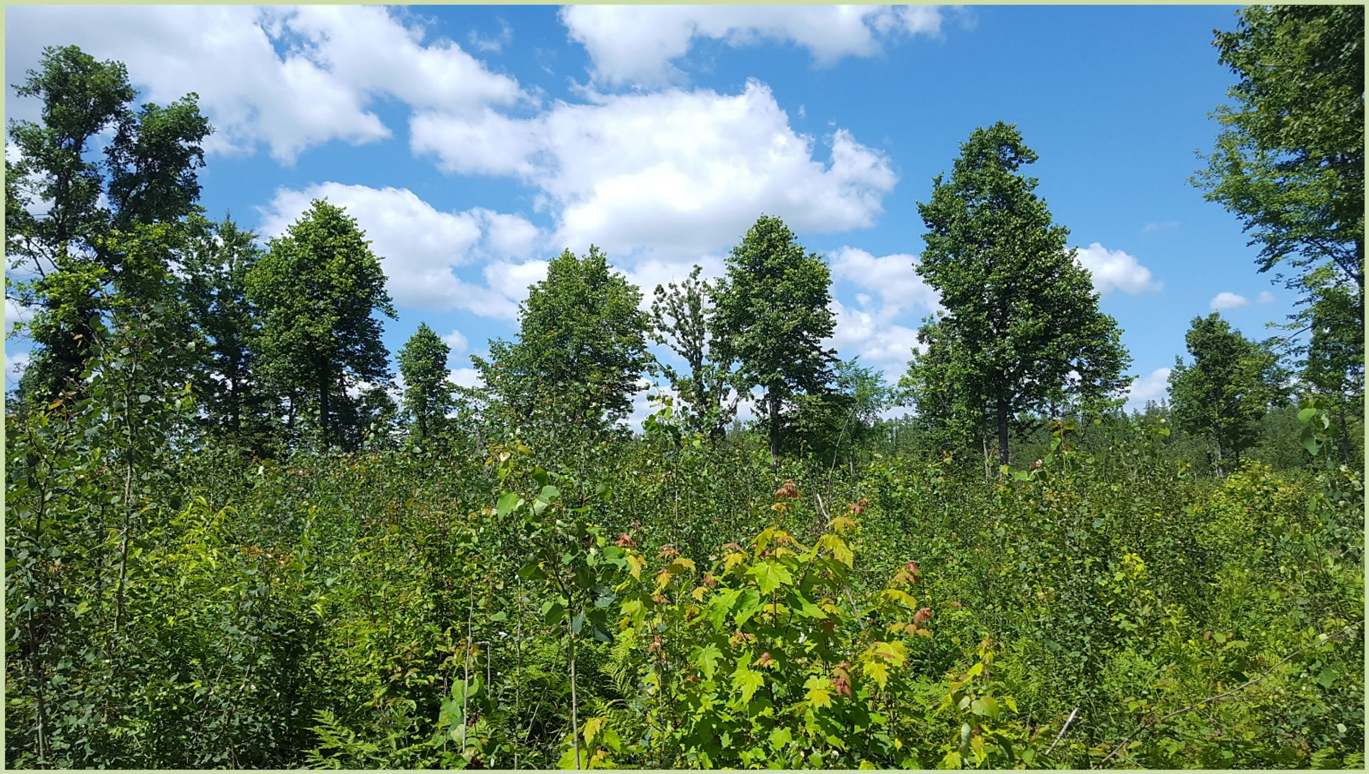

Young forest habitat 3 years after timber harvest

A Conservation Effects Assessment Project (CEAP)

Assessing Wildlife Response to the Natural Resources Conservation Service's (NRCS) Conservation Programs Supporting Young Forest Habitat

Dear [redacted]

We are excited to share with you the results of our second year of Golden-winged Warbler, American Woodcock, and songbird surveys.

As you may know, Golden-winged Warbler and American Woodcock populations have been declining for decades, primarily due to habitat loss in the United States. These two bird species (see cover photos) and many other species of birds, mammals, and reptiles rely on young forest habitat during some or all of their life.

Since 2012, you and 190 other private landowners in 5 different states have created 7,934 acres of young forest habitat through NRCS conservation projects. The critically important young forest habitat you and other participants are creating/have created provides an essential home for an abundant diversity of native wildlife. Together, you are making a positive difference for wildlife in your region!

Your property was visited on 1 occasion (May 19, 2016) to survey for American Woodcock, and visited on 4 occasions (June 3, 2016, June 17, 2016, June 19, 2016 and June 24, 2016) to survey for Golden-winged Warblers and other songbirds. We conducted surveys at 1 point location in the areas where you have created young forest habitat and 4 point locations where you plan to create young forest habitat. Along with this letter we have enclosed a map showing where on your property we surveyed for birds.

On your property, we surveyed American Woodcock in the 1 area that has been managed for young forest. We detected a total of 1 American Woodcock at 1 of 1 points surveyed.

We detected a total of 2 Golden-winged Warblers at 1 of 1 points surveyed in the area that has been managed for young forest. Additionally, we detected a total of 20 species during our surveys in the managed areas (please see below). Moreover, of the 20 species we detected, 3 are currently considered species of greatest conservation need (denoted in the list with an asterisk) so your project is already having meaningful benefits to birds! Along with the list of species, we have included photos of 10 bird species that benefit from young forest on private lands, 9 of which were detected on your property!

1. American Crow
2. American Goldfinch
3. American Redstart
4. American Woodcock*
5. Black-and-white Warbler
6. Black-capped Chickadee
7. Chestnut-sided Warbler
8. Common Loon*
9. Eastern Wood-Pewee
10. Golden-winged Warbler*
11. Hermit Thrush
12. Nashville Warbler
13. Ovenbird
14. Red-eyed Vireo
15. Rose-breasted Grosbeak
16. Turkey Vulture
17. Veery*
18. Wilson's Snipe
19. Wood Duck
20. Yellow-throated Vireo

Thank you for allowing us to conduct these surveys on your property! We look forward to continue working with you to manage and maintain young forest habitat to benefit wildlife like Golden-winged Warblers and American Woodcock on your property and across the region. We are also always looking for more participants, so if anyone you know is interested in forest management opportunities please contact [redacted] (below).

YOUNG FOREST IS ESSENTIAL FOR THESE BIRDS

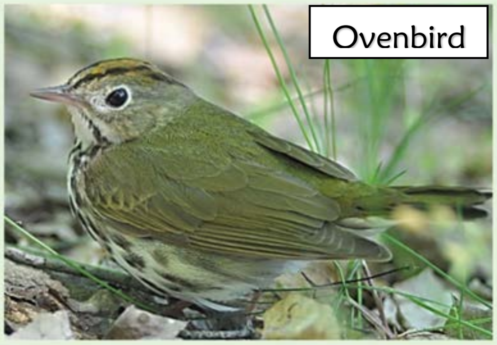

Ovenbird

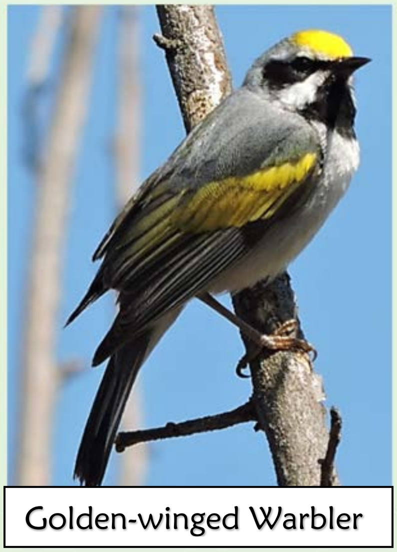

Golden-winged Warbler

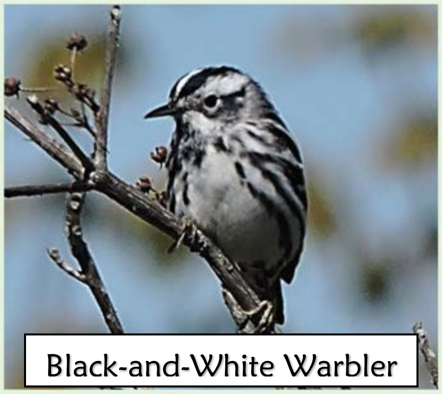

Black-and-White Warbler

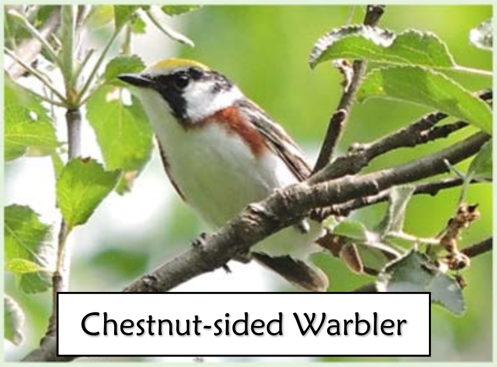

Chestnut-sided Warbler

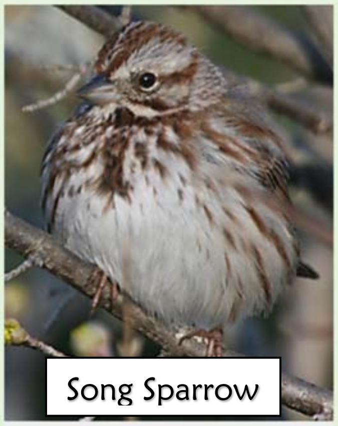

Song Sparrow

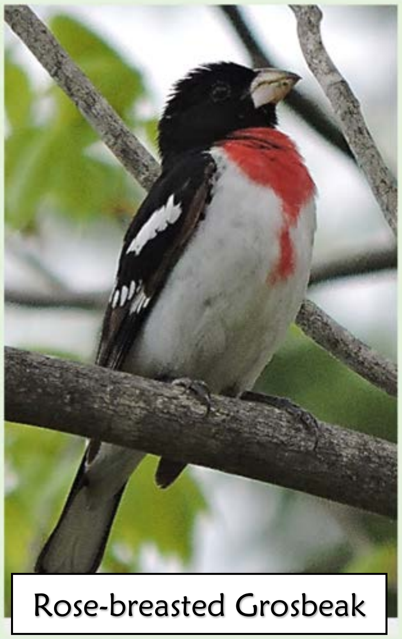

Rose-breasted Grosbeak

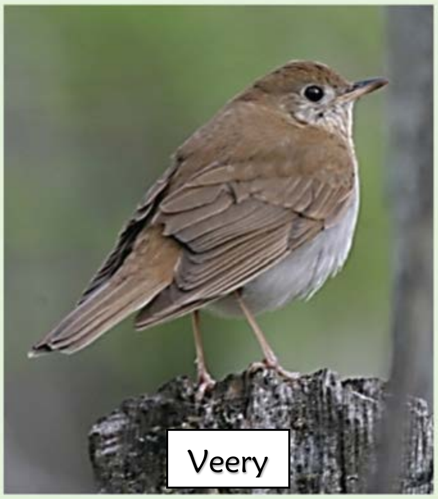

Veery

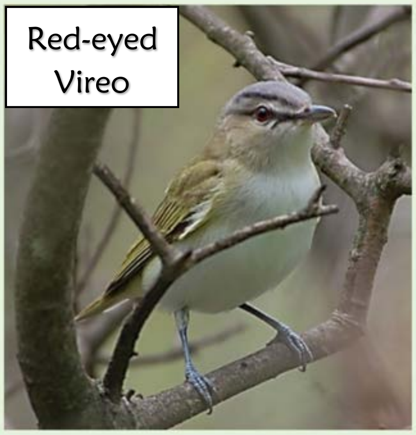

Red-eyed Vireo

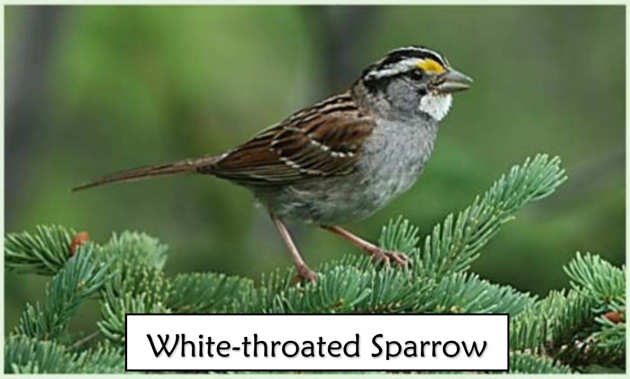

White-throated Sparrow

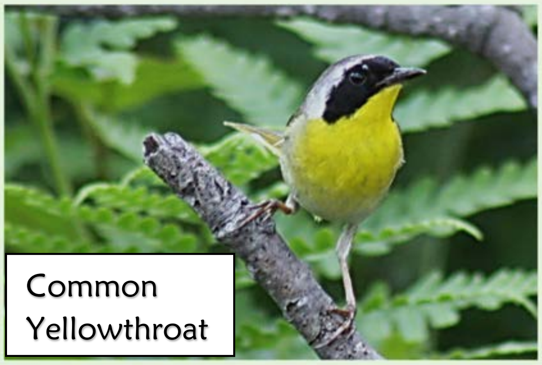

Common Yellowthroat
