# Supplementary material for: Effects of biological monitoring and results outreach on private landowner conservation management
Source: PLoS One. 2018 Apr 4;13(4):e0194740. doi: 10.1371/journal.pone.0194740 (PMC5884542; doi:10.1371/journal.pone.0194740)
Supplement: S2 Appendix — (DOCX) [file pone.0194740.s002.docx]

INTRODUCTION:

Hi, my name is (insert). I am a (affiliation) at [institution]. May I please speak with _______?

If he/she is not available: When may I call back to reach him/her?

Date and time: _______________________

I am conducting a research study to learn more about landowners who enrolled in Natural Resource Conservation Service habitat programs to help improve how well these programs meet conservation goals and landowner objectives for their land. I believe that the NRCS partner biologists let you know I would be calling about your enrolled land. The phone survey will take about 20 to 30 minutes to complete, your participation is voluntary, and your identity and responses will be kept confidential.

Information collected by NRCS partners about your habitat project including land acreage enrolled, contract dates, practices implemented, and biological results of management will be included in this research with your survey responses. The results of this research study will be published and used in a Masters thesis. There are no known risks associated with this survey. Although we would greatly appreciate your help, you are free to decline this survey.

Do you consent to participate in this research study?

Yes 🞏

No 🞏 [if no, thank them and end call].

[If yes] Thanks for your willingness to participate. As we move through the survey, please feel free to ask for clarification if I have phrased something unclearly.

In this survey, I will ask you about your involvement in an NRCS habitat program. Some people participate in more than one NRCS program, so to make it clear which I am talking about, the enrolled property address is (_______) and the contract involved (______) practices.

1. Which category best describes your ownership of the property enrolled in the NRCS habitat program?

Individual 🞏

Joint, such as a husband and wife 🞏

Family partnership 🞏

Trust or Estate 🞏

Club or Association 🞏

Corporation or Business partnership 🞏

**Not an owner** 🞏

Other (please specify) 🞏 _____________

***Responses that classifies respondent as a family forest owner (Individual, Joint, Family Partnership, Trust or Estate) use survey version 1. Responses indicating Club or Association, Corporation or Business, and Other use version 2 of the survey. (Starts on page 11).***

2. Who makes the management decisions, such as whether or not to harvest trees, for the property enrolled in an NRCS habitat program under your name? CHECK ALL THAT APPLY

You 🞏

Your spouse 🞏

Your children 🞏

Another family member 🞏

Your land manager or forester 🞏

Other (please specify) 🞏________________

Next, it would be helpful to know some characteristics of the property you enrolled in the NRCS habitat program.

3. How many years have you or a family member owned the enrolled property for? If you have multiple parcels that were enrolled, think of the one owned longest.

___________

4. About how far do you live from your enrolled property in miles? If you have multiple parcels that were enrolled, think of the nearest.

___________

5. Do you currently have a written property wide forest management or stewardship plan for the enrolled property?

Yes 🞏

No 🞏

NOT SURE 🞏

6. How many acres of land do you own in total?

___________

7. On average, what percentage of your household’s annual income is derived from the wooded land that you own?

___________

___________________________________________________________________________________________

Enrolling land in an NRCS habitat program might result in many different outcomes. We are interested in how important several potential outcomes are for you.

8A. How important to you is having access to expert advice on forest management on your land?

Not at all important 🞏

Slightly important 🞏

Moderately important 🞏

Very important 🞏

Extremely important 🞏

NO RESPONSE 🞏

9A.What effect did participating in the NRCS habitat program have on your access to expert advice on forest management on your land?

Very Positive effect 🞏

Positive effect 🞏

No effect 🞏

Negative effect 🞏

Very negative effect 🞏

8B. How important to you is receiving cost share payments to create or maintain habitat on your land?

9B.What effect did participating in the NRCS habitat program have on your ability to afford costs related to habitat creation or maintenance on your land?

8C. How important to you is improving hunting opportunities on your land?

9C. What effect did participating in the NRCS habitat program have for hunting opportunities on your land?

8D. How important to you is improving bird-watching opportunities on your land?

9D. What effect did participating in the NRCS habitat program have for bird-watching opportunities on your land?

8E. How important to you is benefiting American Woodcock on your land?

9E. What effect did participating in the NRCS habitat program have on American Woodcock on your land?

10A. How certain are you about the answer you just gave?

Very Certain 🞏

Certain 🞏

Neither Certain or Uncertain 🞏

Uncertain 🞏

Very Uncertain 🞏

8F. How important to you is benefiting Golden winged Warblers on your land?

9F. What effect did participating in the NRCS habitat program have on Golden winged Warblers on your land?

10B. How certain are you about the answer you just gave?

8G. How important to you is benefiting other birds that use young forest habitat on your land?

9G. What effect did participating in the NRCS habitat program have on other birds that use young forest habitat your land?

10C. How certain are you about the answer you just gave?

8H. How important to you is improving the scenery on your land?

9H. What effect did participating in the NRCS habitat program have on the scenery on your land?

8I. How important to you is improving the forest health on your land?

9I. What effect did participating in the NRCS habitat program have on the forest health on your land?

8J. How important to you is harvesting timber for income on your land?

9J. What effect did participating in the NRCS habitat program have on timber harvesting for income on your land?

8K. How important to you is increasing property value of your land?

9K. What effect did participating in the NRCS habitat program have on the property value of your land?

_____________________________________________________________________

Okay, now I have a few questions about your level of satisfaction with different parts of the NRCS habitat program. For each you can respond on a 1 to 10 scale, where 1 is not at all satisfied, 5 is moderately satisfied, and 10 is completely satisfied.

11A. How satisfied are you with the conservation program overall?

11B. How satisfied are you with the program cost-share payments?

11C. How satisfied are you with the wildlife outcomes on your land?

11D. How satisfied are you with your interactions with Natural Resources Conservation Service employees, partner biologists, and partner foresters?

Thanks! To what extent do you agree or disagree with the following statements:

12A.You trust the expertise of NRCS employees, partner biologists, and partner foresters to help you achieve your land management goals.

Strongly Agree 🞏

Agree 🞏

Neither 🞏

Disagree 🞏

Strongly Disagree 🞏

NOT SURE 🞏

NO RESPONSE 🞏

12B. You feel that you have similar values to the NRCS employees, partner biologists, and partner foresters.

12C. The rules and procedures of the NRCS habitat program ensure that you are treated fairly.

In the next sections I am going to ask you about managing for young forest on your property. The term young forest refers to areas with well-developed ground cover, shrubs, and young trees, and the absence of a closed tree canopy. It may help to picture the area that you have managed through this NRCS habitat program- how it looks now and over the next few years as the vegetation grows in.

When I say management or managing, I mean taking actions on your property to influence trees or other plant cover. In this survey, when I say managing for young forest I am referring to both actively creating new young forest and taking action to maintain existing young forest.

Your contract with the NRCS habitat program ends in [YEAR] OR ended in [YEAR].

13. How likely are you to manage for (again, as a reminder I mean create or maintain) young forest on your land within ten years after your contract end date by re-enrolling in a Natural Resources Conservation Service program?

Not At All Likely 🞏

Slightly Likely 🞏

Moderately Likely 🞏

Very Likely 🞏

Extremely Likely 🞏

NO RESPONSE 🞏

14. How likely are you to sell or give away any of the forested land you own within ten years after your contract end date?

Not At All Likely 🞏

Slightly Likely 🞏

Moderately Likely 🞏

Very Likely 🞏

Extremely Likely 🞏

NO RESPONSE 🞏

_____________________________________________________________________________________________

**IF CONTRACT HAS ENDED:**

15A. Since your contract ended, have you consulted an expert forester or biologist on habitat management decisions independent of cost share payments?

Yes 🞏

No 🞏

NO RESPONSE 🞏

_____________________________________________________________________________________________

**FOR EVERYONE:**

15B. If further cost share payments were not available how likely are you to consult an expert forester or biologist on future habitat management decisions within ten years after your contract end date?

Not At All Likely 🞏

Slightly Likely 🞏

Moderately Likely 🞏

Very Likely 🞏

Extremely Likely 🞏

NO RESPONSE 🞏

_____________________________________________________________________________________________

**IF CONTRACT HAS ENDED:**

16A. Since your contract ended, have you used any of the following management practices to manage- either create or maintain- for young forest on your land without cost share payments: For each practice you can say yes or no:

Cutting a new patch of trees about 10 acres or more in size

while leaving behind some mature trees standing in the area 🞏

Cutting to expand an existing patch of young forest

while leaving behind some mature trees standing in the area 🞏

Applying herbicides to invasive plants 🞏

Mechanical removal of invasive plants 🞏

Establishing or maintaining native tree or shrub plantings 🞏

Maintaining deer fencing 🞏

Mechanical brush clearing (also called brush hogging) 🞏

Cutting shrubs such as alder 🞏

Prescribed burning 🞏

NOT SURE 🞏

NO RESPONSE 🞏

_____________________________________________________________________________________________

**FOR EVERYONE:**

16B. If further cost share payments were not available, which of the following management practices would you use in the future to manage- either create or maintain- for young forest on your land within ten years after your contract end date: For each practice you can say yes or no:

Cutting a new patch of trees about 10 acres or more in size

while leaving behind some mature trees standing in the area 🞏

Cutting to expand an existing patch of young forest

while leaving behind some mature trees standing in the area 🞏

Applying herbicides to invasive plants 🞏

Mechanical removal of invasive plants 🞏

Establishing or maintaining native tree or shrub plantings 🞏

Maintaining deer fencing 🞏

Mechanical brush clearing (also called brush hogging) 🞏

Cutting shrubs such as alder 🞏

Prescribed burning 🞏

NOT SURE 🞏

NO RESPONSE 🞏

17. If further cost share payments were not available, how likely are you to manage for young forest on your land (using any of the practices described in the previous question) in the future within ten years after your contract end date?

Not At All Likely 🞏

Slightly Likely 🞏

Moderately Likely 🞏

Very Likely 🞏

Extremely Likely 🞏

NO RESPONSE 🞏

18. Now thinking further into the future, if further cost share payments were not available, how likely are you to manage for young forest on your land between ten to twenty years after your contract end date?

Not At All Likely 🞏

Slightly Likely 🞏

Moderately Likely 🞏

Very Likely 🞏

Extremely Likely 🞏

NO RESPONSE 🞏

_____________________________________________________________________________________________

Regardless of whether or not you intend to keep managing for young forest on your land after your contract ends, we are interested in what you think the effects would be if you did choose to manage for young forest without further cost share payments.

To what extent do you agree or disagree with the following statements: (**ONLY ASK ABOUT IMPORTANT OUTCOMES IN QUESTION 8).**

19A. Managing for young forest on your land within ten years after your contract end date would benefit hunting opportunities on your land:

Strongly Agree 🞏

Agree 🞏

Neither Agree or Disagree 🞏

Disagree 🞏

Strongly Disagree 🞏

NOT SURE 🞏

NO RESPONSE 🞏

19B. Managing for young forest on your land within ten years after your contract end date would benefit bird-watching opportunities on your land:

19C. Managing for young forest on your land within ten years after your contract end date would benefit American Woodcock on your land:

19D. Managing for young forest on your land within ten years after your contract end date would benefit Golden-winged Warblers on your land:

19E. Managing for young forest on your land within ten years after your contract end date would benefit other birds that use young forest habitat on your land:

19F. Managing for young forest on your land within ten years after your contract end date would improve the scenery on your land:

19G. Managing for young forest on your land within ten years after your contract end date would benefit forest health on your land:

19H. Managing for young forest on your land within ten years after your contract end date would improve timber harvesting for income on your land:

19I. Managing for young forest on your land within ten years after your contract end date would benefit the property value of your land:

_____________________________________________________________________________________________

20A. How common is it that other landowners in the region purposely manage for young forest on their land without cost share payments?

Not At All Common 🞏

Slightly Common 🞏

Moderately Common 🞏

Very Common 🞏

Extremely Common 🞏

NOT SURE 🞏

NO RESPONSE 🞏

20B. When it comes to management activities you do on your land, how important are the opinions of other landowners in the region to you?

Not at all Important 🞏

Slightly Important 🞏

Moderately Important 🞏

Very Important 🞏

Extremely Important 🞏

NOT SURE 🞏

NO RESPONSE 🞏

To what extent do you agree or disagree with the following statements:

20C. Other landowners in the region think that you should manage your land for young forest within ten years after your contract end date if further cost share payments were not available

Strongly Agree 🞏

Agree 🞏

Neither Agree or Disagree 🞏

Disagree 🞏

Strongly Disagree 🞏

NOT SURE 🞏

NO RESPONSE 🞏

______________________________________________________________________________________________

21A. People who are important to you think that you should manage for young forest on your land within ten years after your contract end date if further cost share payments were not available:

Strongly Agree 🞏

Agree 🞏

Neither Agree or Disagree 🞏

Disagree 🞏

Strongly Disagree 🞏

NOT SURE 🞏

NO RESPONSE 🞏

21B. People you respect and admire think you should manage for young forest on your land within ten years after your contract end date if further cost share payments were not available:

Strongly Agree 🞏

Agree 🞏

Neither Agree or Disagree 🞏

Disagree 🞏

Strongly Disagree 🞏

NOT SURE 🞏

NO RESPONSE 🞏

______________________________________________________________________________________________

22A. If you really wanted to, you could manage for young forest without further cost share payments within ten years after your contract end date:

Strongly Agree 🞏

Agree 🞏

Neither Agree or Disagree 🞏

Disagree 🞏

Strongly Disagree 🞏

NOT SURE 🞏

NO RESPONSE 🞏

22B. For you to continue managing for young forest without further cost-share payments within ten years after your contract end date is under your control:

Strongly Agree 🞏

Agree 🞏

Neither Agree or Disagree 🞏

Disagree 🞏

Strongly Disagree 🞏

NOT SURE 🞏

NO RESPONSE 🞏

______________________________________________________________________________________________

23A. If further cost share payments were not available, is managing for young forest on your land within ten years after your contract end date:

Very Desirable 🞏

Desirable 🞏

Neither Desirable or Undesirable 🞏

Undesirable 🞏

Very Undesirable 🞏

NO RESPONSE 🞏

23B. If further cost share payments were not available, is managing for young forest on your land within ten years after your contract end date:

Very Good 🞏

Good 🞏

Neither Good or Bad 🞏

Bad 🞏

Very Bad 🞏

NO RESPONSE 🞏

______________________________________________________________________________________________

24. If further cost share payments were not available, is your management for young forest on your land within ten years after your contract end date limited by the following factors? For each, you can say yes or no.

You don’t have enough time 🞏

You don’t have enough money 🞏

You don’t know how to manage for young forest 🞏

You don’t have enough acreage 🞏

You don’t like how it looks 🞏

You think that further management

within ten years is unnecessary 🞏

None of those 🞏

NO RESPONSE 🞏

______________________________________________________________________________________________

Okay, that was the final question about how you might manage your land in the future. Now I have a few questions about outdoor recreational behaviors that you may take part in.

25. Do you or a member of your family hunt?

Yes 🞏

No 🞏

NO RESPONSE 🞏

(IF 25 = YES)

26A. Over the past year, about how many days have you personally hunted on the land you enrolled in the NRCS habitat program?

Days _______________________

26B. Over the past year, about how many days have you personally gone hiking or walking on the land you enrolled in the NRCS habitat program?

Days _______________________

26C. Over the past year, about how many days have you personally gone bird-watching on the land you enrolled in the NRCS habitat program?

Days _______________________

26D. Over the past year, about how many days have you personally done other recreational activities on the land you enrolled in the NRCS habitat program?

Days _______________________

______________________________________________________________________________________

27. Since you enrolled in the NRCS habitat program, which of the following people have seen or heard American Woodcock on or near the land you managed for young forest?

You 🞏

NRCS employees, partner foresters, or partner biologists 🞏

Someone else 🞏

NO RESPONSE 🞏

28. Since you enrolled in the NRCS habitat program, which of the following people have seen or heard a Golden-winged Warbler on or near the land you managed for young forest?

You 🞏

NRCS employees, partner foresters, or partner biologists 🞏

Someone else 🞏

NO RESPONSE 🞏

29. Since you enrolled in the NRCS habitat program, which of the following people have seen or heard other birds that use young forest habitat on or near the land you managed for young forest?

You 🞏

NRCS employees, partner foresters, or partner biologists 🞏

Someone else 🞏

NO RESPONSE 🞏

___________________________________________________________________________________

**FOR TREATMENT GROUP ONLY:**

30A. In the past two years you were sent two letters that included bird monitoring results from the enrolled property. Did you receive a mailing last year, which was delivered in October 2015 to you by mail or email?

Yes 🞏

No 🞏

NO RESPONSE 🞏

30B. (IF 30A=YES): Do you recall what this letter said about the presence of birds on your property?

Yes 🞏

No 🞏

NO RESPONSE 🞏

31A. Did you receive a letter in December 2016 by mail which included results from bird monitoring from the enrolled property?

Yes 🞏

No 🞏

NO RESPONSE 🞏

31B. (IF 31A=YES): Do you recall what this letter said about the presence of birds on your property?

Yes 🞏

No 🞏

NO RESPONSE 🞏

______________________________________________________________________________________________

**FOR EVERYONE:**

32A. When biologists visited the enrolled property to monitor for birds did you meet with them?

Yes 🞏

No 🞏

NO RESPONSE 🞏

32B. (IF 32A=YES): Did you accompany these biologists during one or more monitoring site visits?

Yes 🞏

No 🞏

NO RESPONSE 🞏

33. Finally, I would like to ask you some background information about yourself:

Are you a member of any land or wildlife related organizations? Examples might include game species organizations such as the National Wild Turkey Federation, forest owner associations, and conservation organizations such as the Audubon Society.

Yes (specify): _______________________________
None 🞏

NO RESPONSE: 🞏

34. In what year were you born?

Year: ___________________

NO RESPONSE: 🞏

35. What is the highest level of formal education you have completed?

Less than high school 🞏

High school diploma/G.E.D 🞏

Some college or technical school 🞏

Associate’s degree 🞏

College undergraduate degree 🞏

(for example, B.A., B.S.)

Graduate or professional degree 🞏

(for example, M.S., Ph.D., M.D.)

NO RESPONSE 🞏

36. What is your gender?

Male 🞏

Female 🞏

Other 🞏

NO RESPONSE 🞏

______________________________________________________________________________________________

**FOR CONTROL GROUP ONLY:**

37. Thanks so much! A letter with the monitoring results from your property will be arriving in the next month. Would you be willing to take a shorter follow up phone survey if I called you back sometime in May?

Yes 🞏

No 🞏

_____________________________________________________________________________________________

That is all the questions that I have for you. Thank you for your time and have a great day.

VERSION 2 for Group Ownership Respondents

2. Who makes the management decisions, such as whether or not to harvest trees, for the property enrolled in an NRCS habitat program under your name? CHECK ALL THAT APPLY

You 🞏

Your spouse 🞏

Your children 🞏

Another family member 🞏

Your land manager or forester 🞏

Other (please specify) 🞏________________

Next, it would be helpful to know some characteristics of the property you enrolled in the NRCS habitat program.

3. How many years has your group owned the enrolled property for? If the group had multiple parcels that were enrolled, think of the one owned longest.

___________

4. About how far do you live from the enrolled property in miles? If the group had multiple parcels that were enrolled, think of the nearest.

___________

5. Does the group currently have a written property wide forest management or stewardship plan for the enrolled property?

Yes 🞏

No 🞏

NOT SURE 🞏

6. How many acres of land does the group own in total?

___________

7. On average, what percentage of your group’s annual income is derived from the wooded land that they own?

___________

_____________________________________________________________________________________________

Enrolling land in an NRCS habitat program might result in many different outcomes. We are interested in how important several potential outcomes are for your group.

8A. How important to your group is having access to expert advice on forest management on their land?

Not at all important 🞏

Slightly important 🞏

Moderately important 🞏

Very important 🞏

Extremely important 🞏

NO RESPONSE 🞏

9A. What effect did participating in the NRCS habitat program have on your group’s access to expert advice on forest management on their land?

Very Positive effect 🞏

Positive effect 🞏

No effect 🞏

Negative effect 🞏

Very negative effect 🞏

8B. How important to your group is receiving cost share payments to create or maintain habitat on their land?

9B. What effect did participating in the NRCS habitat program have on your group’s ability to afford costs related to habitat creation or maintenance on their land?

8C. How important to your group is improving hunting opportunities on their land?

9C. What effect did participating in the NRCS habitat program have for hunting opportunities on the group’s land?

8D. How important to your group is improving bird-watching opportunities on their land?

9D. What effect did participating in the NRCS habitat program have for bird-watching opportunities on the group’s land?

8E. How important to your group is benefiting American Woodcock on their land?

9E. What effect did participating in the NRCS habitat program have on American Woodcock on the group’s land?

10A. How certain are you about the answer you just gave?

Very Certain 🞏

Somewhat Certain 🞏

Neither Certain or Uncertain 🞏

Somewhat Uncertain 🞏

Very Uncertain 🞏

8F. How important to your group is benefiting Golden winged Warblers on their land?

9F. What effect did participating in the NRCS habitat program have on Golden winged Warblers on the group’s land?

10B. How certain are you about the answer you just gave?

8G. How important to your group is benefiting other birds that use young forest on their land?

9G. What effect did participating in the NRCS habitat program have on other birds that use young forest on the group’s land?

10C. How certain are you about the answer you just gave?

8H. How important to your group is improving the scenery on their land?

9H. What effect did participating in the NRCS habitat program have on the scenery on the group’s land?

8I. How important to your group is improving the forest health on their land?

9I. What effect did participating in the NRCS habitat program have on the forest health on the group’s land?

8J. How important to your group is harvesting timber for income on their land?

9J. What effect did participating in the NRCS habitat program have on timber harvesting for income on the group’s land?

8K. How important to your group is increasing property value of their land?

9K. What effect did participating in the NRCS habitat program have on the property value of the group’s land?

_____________________________________________________________________

Okay, now I have a few questions about your group’s level of satisfaction with different parts of the NRCS habitat program. For each you can respond on a 1 to 10 scale, where 1 is not at all satisfied, 5 is moderately satisfied, and 10 is completely satisfied.

11A. How satisfied is the group with the conservation program overall?

11B. How satisfied is the group with the program cost-share payments?

11C. How satisfied is the group with the wildlife outcomes on their land?

11D. How satisfied is the group with their interactions with Natural Resources Conservation Service employees, partner biologists, and partner foresters?

Thanks! To what extent do you agree or disagree with the following statements.

12A.Your group trusts the expertise of NRCS employees, partner biologists, and partner foresters to help them achieve their land management goals.

Strongly Agree 🞏

Agree 🞏

Neither 🞏

Disagree 🞏

Strongly Disagree 🞏

NOT SURE 🞏

NO RESPONSE 🞏

12B. Your group feels that they have similar values to the NRCS employees, partner biologists, and partner foresters.

12C. The rules and procedures of the NRCS habitat program ensure that your group is treated fairly.

In the next sections I am going to ask you about managing for young forest on your property. The term young forest refers to areas with well-developed ground cover, shrubs, and young trees, and the absence of a closed tree canopy. It may help to picture the area that you have managed through this NRCS habitat program- how it looks now and over the next few years as the vegetation grows in.

When I say management or managing, I mean taking actions on your property to influence trees or other plant cover. In this survey, when I say managing for young forest I am referring to both actively creating new young forest and taking action to maintain existing young forest.

Your contract with the NRCS habitat program ends in [YEAR] OR ended in [YEAR].

11. How likely is your group to manage for (again, as a reminder I mean create or maintain) young forest on their land within ten years after the contract end date by re-enrolling in a Natural Resources Conservation Service program?

Not At All Likely 🞏

Slightly Likely 🞏

Moderately Likely 🞏

Very Likely 🞏

Extremely Likely 🞏

NO RESPONSE 🞏

12. How likely is your group to sell or give away any of the forested land they own within ten years after the contract end date?

Not At All Likely 🞏

Slightly Likely 🞏

Moderately Likely 🞏

Very Likely 🞏

Extremely Likely 🞏

NO RESPONSE 🞏

_____________________________________________________________________________________________

**IF CONTRACT HAS ENDED**

13A. Since the contract ended, has your group consulted an expert forester or biologist on habitat management decisions independent of cost share payments?

Yes 🞏

No 🞏

NO RESPONSE 🞏

_____________________________________________________________________________________________

**FOR EVERYONE**

13B. If further cost share payments were not available how likely is your group to consult an expert forester or biologist on future habitat management decisions within ten years after the contract end date?

Not At All Likely 🞏

Slightly Likely 🞏

Moderately Likely 🞏

Very Likely 🞏

Extremely Likely 🞏

NO RESPONSE 🞏

_____________________________________________________________________________________________

**IF CONTRACT HAS ENDED**

14A. Since the contract ended, has your group used any of the following management practices to manage- either create or maintain- for young forest on their land without cost share payments: For each practice you can say yes or no:

Cutting a new patch of trees about 10 acres or more in size

while leaving behind some mature trees standing in the area 🞏

Cutting to expand an existing patch of young forest

while leaving behind some mature trees standing in the area 🞏

Applying herbicides to invasive plants 🞏

Mechanical removal of invasive plants 🞏

Establishing or maintaining native tree or shrub plantings 🞏

Maintaining deer fencing 🞏

Mechanical brush clearing (also called brush hogging) 🞏

Cutting shrubs such as alder 🞏

Prescribed burning 🞏

NOT SURE 🞏

NO RESPONSE 🞏

_____________________________________________________________________________________________

**FOR EVERYONE**

14B. If further cost share payments were not available, which of the following management practices would your group use in the future to manage- either create or maintain- for young forest on their land within ten years after the contract end date: For each practice you can say yes or no:

Cutting a new patch of trees about 10 acres or more in size

while leaving behind some mature trees standing in the area 🞏

Cutting to expand an existing patch of young forest

while leaving behind some mature trees standing in the area 🞏

Applying herbicides to invasive plants 🞏

Mechanical removal of invasive plants 🞏

Establishing or maintaining native tree or shrub plantings 🞏

Maintaining deer fencing 🞏

Mechanical brush clearing (also called brush hogging) 🞏

Cutting shrubs such as alder 🞏

Prescribed burning 🞏

NOT SURE 🞏

NO RESPONSE 🞏

15. If further cost share payments were not available, how likely is your group to manage for young forest on their land (using any of the practices described in the previous question) in the future within ten years after the contract end date?

Not At All Likely 🞏

Slightly Likely 🞏

Moderately Likely 🞏

Very Likely 🞏

Extremely Likely 🞏

NO RESPONSE 🞏

16. Now thinking further into the future, if further cost share payments were not available, how likely are you to manage for young forest on your land between ten to twenty years after your contract end date?

Not At All Likely 🞏

Slightly Likely 🞏

Moderately Likely 🞏

Very Likely 🞏

Extremely Likely 🞏

NO RESPONSE 🞏

_____________________________________________________________________________________________

24. If further cost share payments were not available, is your group’s management for young forest on their land within ten years after the contract end date limited by the following factors? For each, you can say yes or no.

Your group does not have enough time 🞏

Your group does not have enough money 🞏

Your group does not know how to manage for young forest 🞏

Your group does not have enough acreage 🞏

Your group does not like how it looks 🞏

Your group thinks that further management

within ten years is unnecessary 🞏

None of those 🞏

NO RESPONSE 🞏

______________________________________________________________________________________

27. Since your group enrolled in the NRCS habitat program, which of the following people have seen or heard American Woodcock on or near the land your group managed for young forest?

You 🞏

NRCS employees, partner foresters, or partner biologists 🞏

Someone else 🞏

NO RESPONSE 🞏

28. Since your group enrolled in the NRCS habitat program, which of the following people have seen or heard a Golden-winged Warbler on or near the land your group managed for young forest?

You 🞏

NRCS employees, partner foresters, or partner biologists 🞏

Someone else 🞏

NO RESPONSE 🞏

29. Since you enrolled in the NRCS habitat program, which of the following people have seen or heard other birds that use young forest habitat on or near the land you managed for young forest?

You 🞏

NRCS employees, partner foresters, or partner biologists 🞏

Someone else 🞏

NO RESPONSE 🞏

_____________________________________________________________________________________________

**FOR TREATMENT GROUP ONLY:**

30A. In the past two years your group was sent two letters that included bird monitoring results from the enrolled property. Did your group receive a mailing last year, which was delivered in October 2015 by mail or email?

Yes 🞏

No 🞏

NO RESPONSE 🞏

30B. (IF 30A=YES): Do you recall what this letter said about the presence of birds on your property?

Yes 🞏

No 🞏

NO RESPONSE 🞏

31A. Did your group receive a letter in December 2016 by mail which included bird monitoring results from your property?

Yes 🞏

No 🞏

NO RESPONSE 🞏

31B. (IF 31A=YES): Do you recall what this letter said about the presence of birds on your property?

Yes 🞏

No 🞏

NO RESPONSE 🞏

______________________________________________________________________________________________

**FOR EVERYONE:**

32A. When biologists visited the enrolled property to monitor for birds did you meet with them?

Yes 🞏

No 🞏

NO RESPONSE 🞏

32B. (IF 32A=YES): Did you accompany these biologists during one or more monitoring site visits?

Yes 🞏

No 🞏

NO RESPONSE 🞏

34. In what year were you born?

Year: ___________________

NO RESPONSE: 🞏

35. What is the highest level of formal education you have completed:

Less than high school 🞏

High school diploma/G.E.D 🞏

Some college or technical school 🞏

Associate’s degree 🞏

College undergraduate degree 🞏

(for example, B.A., B.S.)

Graduate or professional degree 🞏

(for example, M.S., Ph.D., M.D.)

NO RESPONSE 🞏

36. What is your gender?

Male 🞏

Female 🞏

Other 🞏

NO RESPONSE 🞏

___________________________________________________

**FOR CONTROL GROUP ONLY:**

37. Thanks so much! A letter with the monitoring results from your property will be arriving in the next month. Would you be willing to take a short follow up phone survey if I called you back sometime in May?

Yes 🞏

No 🞏

_____________________________________________________________________________________________

That is all the questions that I have for you. Thank you for your time and have a great day!
